# Epigenetics of cardiomyopathies: the next frontier

**DOI:** 10.1007/s10741-024-10460-4

**Published:** 2024-11-26

**Authors:** Aida Hajdarpašić, Martijn Tukker, Wouter te Rijdt, Sharida Mohamedhoesein, Wouter C. Meijers, Kadir Caliskan

**Affiliations:** 1 Department of Medical Biology and Genetics, Sarajevo Medical School, University Sarajevo School of Science and Technology, Hrasnička Cesta 3a, 71210 Sarajevo, Ilidža Bosnia and Herzegovina; 2 Thoraxcenter, Department of Cardiology, Cardiovascular Institute. Erasmus MC - University Medical Center Rotterdam, Office RG-431Dr. Molewaterplein 40, 3015 GD Rotterdam, The Netherlands; 3 Department of Clinical Genetics, Erasmus MC University Medical Center, Rotterdam, The Netherlands

**Keywords:** Cardiomyopathy, Epigenetics, Phenotype, Environment, Clinical expression, Genotype

## Abstract

Cardiomyopathies (CMP) are a diverse group of myocardial diseases that cause structural, functional, and pathological changes to the heart. Alterations at the molecular level associated with the clinical phenotype and progression of CMPs cannot be solely explained by the genetic mutations, even in inherited cardiomyopathies. Epigenetics and environmental factors are likely to significantly modify the clinical manifestations of CMPs, resulting in variable clinical expression and different age-related penetrance. This review examines the role of dysfunctional DNA methylation, histone modifications, chromatin remodelling, and noncoding RNAs in the development and exacerbation of CMPs, highlighting their potential as diagnostic markers and therapeutic targets, including the use of histone deacetylase inhibitors. Additionally, it explores how environmental exposures can influence epigenetic changes and potentially be used for preventive strategies and personalized care in CMP patients. Monozygotic twin studies and intergenerational studies are discussed as valuable tools for understanding the interplay between genetics, epigenetics, and environmental factors. Lastly, this review addresses current challenges and future perspectives, such as the need for greater specificity in epigenetic therapies, minimizing off-target effects, and investigating sex differences in CMP research and treatment.

**Supplementary Information:**

The online version contains supplementary material available at 10.1007/s10741-024-10460-4.

## Introduction

**Fig. 1:**
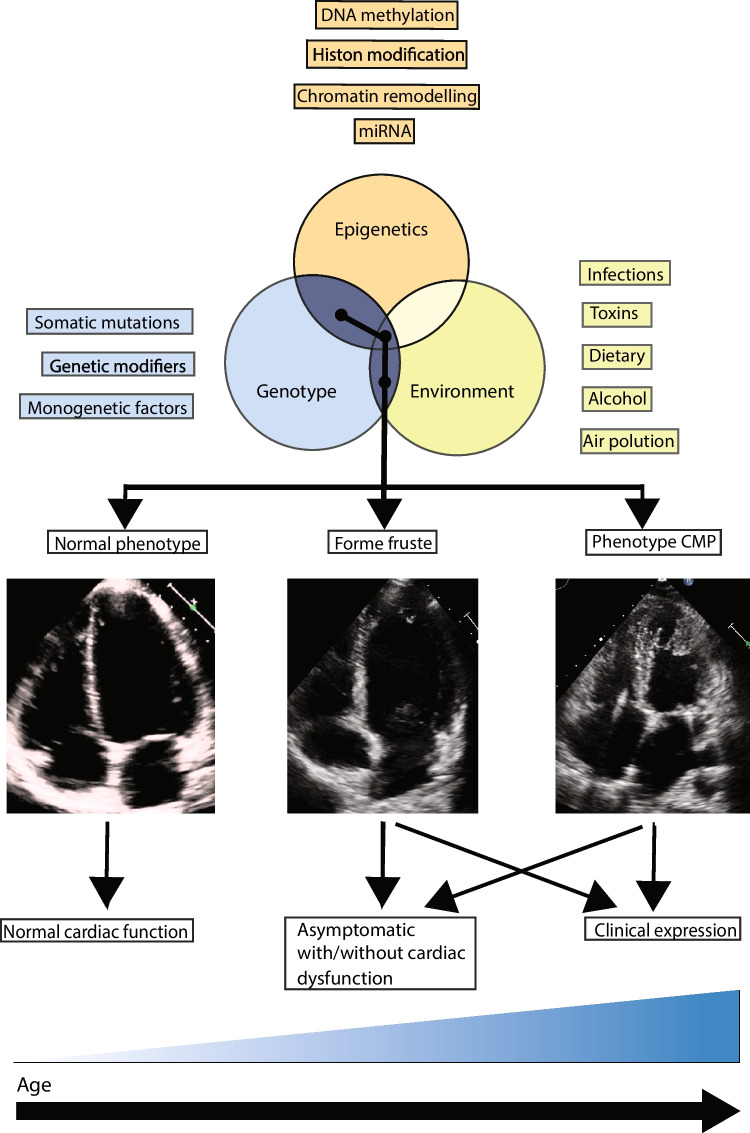
Interplay between genotype, epigenome, and environmental factors resulting in different phenotypes

Cardiomyopathies (CMP) are a heterogeneous group of myocardial diseases, characterized by structural and functional pathological changes in cardiac tissue. CMP pose a significant health burden, as it can manifest as advanced heart failure (HF), arrhythmia, and sudden cardiac death (SCD) [1, 2]. The majority of current research on cardiomyopathies is focused on genetics, as gene variants can cause cardiomyopathies, and genetic testing is a standard clinical practise. Gene variants offer valuable insights and clinical actionability, including insights into the etiology, early detection in (asymptomatic) family members, and potential preventive measures or therapies based on the type of gene variant for CMP. However, alterations at the molecular level associated with the clinical phenotype, expression, and progression of CMPs are not solely explained by the genetic mutations. Other mechanisms such as epigenetics and environmental factors are likely to play a significant role in the clinical manifestations (Fig. 1).

**Fig. 2:**
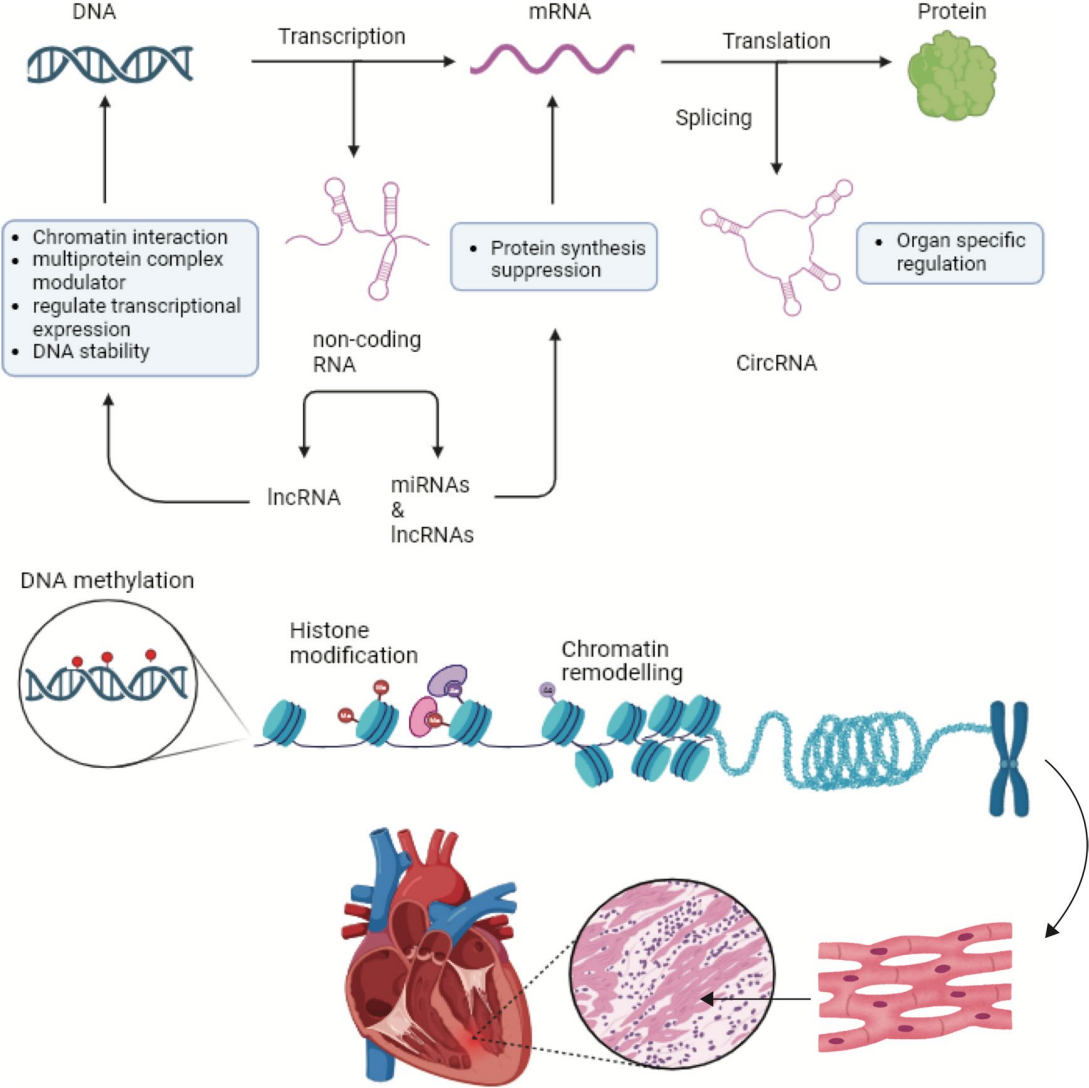
Epigenetic mechanisms DNA methylation, histone modification, chromatin remodeling, and non-coding RNAs regulating the onset and/or progression of cardiomyopathies. This figure was created using BioRender

The term “epigenome” is used to describe marks on the genome that influence the phenotype of individuals without altering the underlying deoxyribonucleic acid (DNA) sequence. The epigenetic regulation of the genome is mediated by modifications such as DNA methylation, histone modification, chromatin remodeling, and post-transcriptional mechanisms such as microRNAs (miRNAs), as well as other non-coding RNAs such as long non-coding RNAs (lncRNAs) and circular RNAs (circRNAs) (Fig. 2). Movassagh et al. have identified distinct epigenomic patterns in important DNA elements of the cardiac genome in human end-stage cardiomyopathy, suggesting that the epigenome plays a crucial role in controlling the expression of genes involved in myocardial stress response, both locally and distally [3]. One example of epigenetic regulation is global changes in DNA methylation, which have been linked to the onset of various cardiovascular diseases [4]. DNA methylation involves the addition of a methyl group to cytosine within CpG dinucleotides without altering the DNA sequence, a process facilitated by DNA methyltransferase (DNMT). There are 3 classes of DNMTs, with DNMT1 and DNMT3 playing the most important roles. DNMT3a and DNMT3b are responsible for de novo methylation, whereas DNMT1 is ubiquitously expressed and has the primary function of maintaining DNA methylation. The methylation of DNA in repetitive sequences helps maintain chromosome stability during cell division, represses transposon expression in mammalian cells, and is involved in embryonic development, X chromosome inactivation, and genomic imprinting [5]. Aberrant DNA methylation has been linked to the development of many diseases, including cardiomyopathies.

Histone proteins form the building blocks of nucleosomes, which are the fundamental units of chromatin. The amino acid residues of histone proteins, particularly those at the N-terminus, undergo post-translational modifications, including acetylation, methylation, phosphorylation, SUMOylation and ubiquitylation [6]. Acetylation of histone tails by histone acetyltransferases (HATs) neutralizes the histone’s positive charge, leading to the loosening of nucleosomes. This decompaction makes DNA more accessible to RNA polymerases and transcription factors, promoting transcription. In contrast, histone deacetylases (HDACs) remove acetyl groups, leading to compaction and transcriptional repression [7]. While compacted and stable nucleosome structure protects DNA sequence, it also restricts its accessibility. Therefore, in order to modulate DNA access, chromatin remodeling complexes alter the chromatin structure by changing nucleosome positioning and loosening the DNA to histone contacts, thereby enabling access of different proteins to DNA for replication, repair, recombination, or transcription [8].

MiRNAs play an important role in post-transcriptional regulation. These short (~ 20 base pairs long), single-stranded, non-coding RNA molecules directly regulate gene expression, affecting a wide range of cellular functions. MiRNAs suppress protein synthesis by base pairing with specific regions of messenger RNAs (mRNAs), primarily the 3′untranslated region (3′UTR), though base pairing to the 5′UTR and coding regions have also been reported [9]. Another type of non-coding RNA linked to the development of cardiomyopathies is lncRNA. LncRNAs are over 200 nucleotides in length, do not encode proteins, and can undergo splicing, capping, and polyadenylation. They are found in both the nucleus and cytoplasm and are involved in various biological processes, including: (a) interacting with chromatin complexes to regulate gene expression epigenetically, (b) modulating proteins or multiprotein complexes, (c) binding to DNA/RNA-associated proteins to control transcription, (d) maintaining DNA stability through R-loop and triple helix formation, and (e) contributing to the formation of higher-order chromatin structures [10].

Finally, circular RNAs can be categorized based on their splicing mechanisms and the presence of exons or introns. In addition to originating from RNA lariats, back splicing is a major mechanism for generating circRNAs [11]. Some circRNAs are specific to certain organs. For example RBM20, a critical splicing factor essential for the normal splicing of numerous cardiac genes, is known to regulate the formation of circRNAs from skipped exons in the I-band region of the Titin gene. The absence of RBM20 is associated to early-onset dilated cardiomyopathy (DCM) [12, 13]. A recent study found that there was overlap between circRNAs regulated by RBM20 and those regulated by the RNA-binding protein quaking (QKI). Interestingly many of these overlapping circRNAs are derived from the Titin gene and are regulated in opposing ways [14]. This suggests that organ-specific circRNAs can be specific to a single gene, highlighting the importance of organ-specific biogenesis and function of circRNAs [15, 16].

Current guidelines highlight that epigenetic factors, along with other acquired modifiers such as pregnancy and hypertension, may aggravate or trigger the DCM phenotype. These guidelines stress the importance of considering the interaction between genetic predisposition and acquired factors during the diagnostic work-up. However, they do not yet specify particular methodologies or epigenetic markers, as the nature of these markers remains largely unknown. This underscores the need for future research to develop standardized protocols and recommendations in this area [17].

This review synthesizes existing literature and addresses knowledge gaps in the field of epigenetics in cardiomyopathies, focussing on the interplay between genetic and epigenetic mechanisms, environmental exposures, and the current evidence from twin studies. It also explores experimental models and emerging epigenetic next-generation therapies, which may offer a novel therapeutic approach for cardiomyopathies, for which innovations have been scarce in the last few decades.

## Epigenetics in development of cardiomyopathies

Alterations in DNA methylation profiles of cardiac tissue have been linked to the development of DCM (Table S1**A**) [18]. As a result, researchers are investigating their potential use as cardiomyopathy biomarkers. Notably, methylation markers are able to discriminate patients with Chagas cardiomyopathy from asymptomatic individuals with 95% precision and predict the severity of the disease, based on heart ejection fraction rate, with 96% precision [18]. Additionally, significant differences in gene expression and DNA methylation profiles have been observed in the left ventricle (LV) myocardial samples from patients with hypertrophic cardiomyopathy (HCM) compared to those with aortic stenosis (AS) [19]. These findings highlight distinct molecular mechanisms that underlie primary and secondary myocardial hypertrophy. Moreover, in DCM hearts, the active regulatory element H3K27ac and its associated connectome (H3K27ac loops) are extensively reprogrammed, contributing to transcriptional dysregulation involved in DCM progression [20]. Above-mentioned evidence points to significant involvement of these epigenetic mechanisms in development of cardiomyopathies.

Histone methyltransferases (HMTs) and histone demethylases (HDMs) have been highly implicated in pathophysiological onset of cardiomyopathies as well (Table S1**B**) [21]. For instance, the p300 histone acetyltransferase acts as a transcriptional coactivator by bridging DNA-binding transcription factors with the basal transcriptional machinery and serving as a scaffold for other transcription factors [21]. Comparative studies of histone modifications in the left- and right-ventricles (RV) at the level of atrial natriuretic peptide (ANP) and brain natriuretic peptide (BNP) have shown higher levels of activating modifications, such as H3K4me2 and H3K9ac, in the LV, accompanied by higher expression of ANP and BNP compared to the RV [19]. In failing LV, upregulation of both ANP and BNP has been observed, with a reduction in repressive modifications like H3K9me2 and H3K9me3 [22]. These aberrantly regulated genes could be potential targets for further studies of epigenetic regulation in cardiomyopathies. ChIP-seq analysis, targeting histone modifications associated with active transcriptional enhancers, was conducted on LV tissue, showing that pathological heart conditions are not characterized by a general disruption of gene expression [23]. Instead, they involve a specific and complex genome-wide shift in transcriptional regulation, particularly affecting a distinct set of distant-acting enhancers [23].

Another epigenetic mechanism involved in cardiomyopathies is chromatin remodeling (Table S1**C**). Four different families of ATP-dependent chromatin remodeling complexes have been described so far: Switch/Sucrose Nonfermentable complex (SWI/SNF), imitation switch complexes (ISWI), chromodomain–helicase–DNA-binding complexes (CHD) and inositol-requiring 80 (INO80) complexes [24, 25]. All members of each family show peculiar DNA-histone contacts domains for chromatin remodeling, thereby addressing specific cellular need by precisely regulating gene expression [26].Transcriptional activators promote an open, accessible chromatin structure, allowing the recruitment of transcription factors that positively regulate gene expression. One such family of proteins is the SWI/SNF complex, which promotes the formation of open-conformation chromatin [27, 28]. Although the clinical relevance of SWI/SNF mutations has traditionally been associated with their role in tumor suppression, studies of the past decade have shed a light on its critical role in cardiovascular development. The SWI/SNF complex, containing BRG1 (Brahma-related gene 1) as its catalytic ATPase subunit, interacts with cardiogenic transcription factors critical to cardiac development (e.g. TBX5, GATA-4 and NKX2-5) [29, 30]. Interestingly, a recent study showed that BRG1 maintains cardiomyocyte homeostasis by regulating cardiomyocyte mitophagy and mitochondrial dynamics, implicating its importance in both cardiovascular development and repair [29]. However, once activated by stress, BRG1 orchestrates the epigenetic control by recruiting G9a and DNMT3 to catalyze the chromatin methylation on Myh6 promoter, resulting in the silencing of the gene and in impaired cardiac contraction [31]. Another element of the SWI/SNF complex is DPF3, a transcription regulator that binds acetylated histones enabling a regulatory switch between poised and activated chromatin stages [32]. Following the interaction between BRG1 and the activated DFP3a, the transcriptional repressor is released from the DNA and the transcription of the downstream targets is activated, which in turn results in pathological cardiac hypertrophy [33].

Finally, RNA modification results in post-transcriptional regulation, mainly by regulating mRNA stability and translation. RNA modifying enzymes can be classified as writers, readers and erasers. N6-methyladenosine (m6A) is one of the abundant mRNA modifications, governing RNA processing by recognizing m6A binding proteins [34]. In patients with HF with preserved ejection fraction compared to healthy controls, a significantly altered expression pattern with upregulation of m6A writers (*METTL3, METTL4 and KIAA1429*), eraser *FTO*, and reader *YTHDF2* was found [35]. In an in vitro experiment with primary cardiomyocytes, METTL3-mediated methylation of mRNA N^6^-adenosines was enhanced in response to hypertrophic stimuli and was essential for a normal hypertrophic response [36]. Additionally, in a murine model, increased m6A RNA methylation caused compensated hypertrophy, whereas a decrease resulted in cardiomyocyte remodeling and dysfunction. While research into RNA modification in CMP is still ongoing, these alterations in the landscape of m6A enzymes might present an interesting target for therapeutic interventions.

So, to deepen the understanding of epigenetic factors in cardiomyopathy, both in vivo and in vitro experimental models are being explored.

An important aspect to keep in mind when using animal models, however, is the lack of conservation across species of epigenetic factors. The use of induced pluripotent stem cells (iPSCs) aids in finding the human equivalent of the epigenetic factors and allows for further research. For example, a study which performed transcriptome sequencing of iPSC cardiomyocytes at different stages was able to identify epigenetic factors crucial for normal cardiac development and healthy cardiomyocyte biology [37].

## Noncoding RNAs in cardiomyopathies

Non-coding RNAs have been explored in correlation to CMP as both potential biomarkers, as discussed in this section, and as potential therapeutic targets or modulators, which is discussed in the section on new therapies targeting epigenetics (Table S1**D)**. Causative mutations of arrhythmogenic cardiomyopathy (ACM) have been shown to affect miRNA profiling in cardiac tissue. A recent study used two transgenic murine models carrying mutations found in ACM patients. Not only did these mice present with decreased Wnt/β-catenin signalling, but genome-wide RNA-sequencing showed upregulation of miR-217-5p and miR-708-5p, along with a downregulation in miR-499-5p [38]. Therefore, these novel miRNAs may turn out to be important new biomarkers in future studies of epigenetic regulation in ACM patients. A recent study screened for differentially expressed miRNAs to functionally characterize their effects in cardiac mesenchymal progenitor cells, aiming to further validate the ACM phenotype [39]. This study found that PKP2 deficiency suppresses the E2F1 pathway, leading to hypermethylation of the miR-184 promoter [39]. This hypermethylation results in decreased miR-184 levels, contributing to the onset of adipogenesis and providing yet another potential epigenetic target for future studies. Moreover, another study screened 750 miRNAs and identified 59 that were significantly modulated in plakophilin 2-deficient HL-1 cells, with mir-184 being the most strongly regulated in this context [21]. The potential use of miRNAs as diagnostic tools for various pathophysiology’s has gained much interest over the last decade [40]. One pivotal study found a correlation between ACM and low plasma levels of miR-320a, a miRNA implicated in the pathogenesis of ACM [41]. This miRNA was shown increase during adipogenic differentiation of human mesenchymal bone marrow cells [42] and was found to regulate the Wnt pathway in human colon cancer cells [40]. Besides miRNAs, lncRNA and circRNA are also recognized for their roles in regulating various cellular processes. This includes mediating different physiological and pathological processes through miRNA sponging, regulating mRNA translation, transcription silencing and reprogramming epigenetic processes [43]. Dysregulated global expression patterns of lncRNA have been described in cardiovascular conditions. Recent research has identified specific lncRNAs involved in cardiomyopathies, including lncDACH1 (Dachshund Homolog 1), which was found to regulate cardiac function in mice by inhibiting SERCA2a [44]. This reduces calcium transients and cell shortening leading to cardiac dysfunction and CMP. An upregulation of lncDAH1 was also found in cardiac tissue samples from patients with CMP compared to nonfailing hearts, indicating that the same mechanism leading to cardiac dysfunction could take place in humans.

On the other hand, recent studies have found that lncRNA KCND1 (lncKCND1) plays a protective role in preventing cardiac hypertrophy [45]. Further experiments revealed that Y-box binding protein 1 (YBX1) can directly bind to lncKCND1, and its expression levels closely mirror those of lncKCND1. More importantly, silencing of YBX1 reversed the protective effect of lncKCND1, suggesting that YBX1 is a downstream target in the machinery of lncKCND1 regulation of cardiac hypertrophy [45]. Interestingly, some lncRNAs appear to exhibit cell type-specific cardiac pathophysiological properties. LncRNA-chast (cardiac hypertrophy-associated transcript) has been shown to regulate cardiac hypertrophy at the cardiomyocyte level [46]. Similarly, lncRNA-wisper (Wisp2 super-enhancer-associated RNA) has been identified as a cardiac fibroblast-enriched lncRNA, regulating fibrosis after cardiac injury and correlating with fibrosis in both mice and human hearts with aortic stenosis [47].

CircRNAs are noncoding RNAs generated from RNA transcripts through non-canonical back-splicing events that connect exon boundaries inversely [43]. They act as scavengers to capture other RNA molecules, altering or controlling expression levels of other regulatory proteins or RNAs. The newly identified conserved circRNA DICAR (diabetes-induced circulation-associated circular RNA) was found to efficiently inhibit DCM [48]. Mouse models showed that DICAR insufficiency led to spontaneous cardiac dysfunction, hypertrophy, and cardiac fibrosis, whereas overexpression of DICAR alleviated DCM. Additionally, expression levels of DICAR in circulating blood cells, plasma and cardiac tissue were lower in diabetic patients than healthy controls [48]. From diagnostic point of view, circulating lncRNAs XLOC015617, AK035192, Gm10435, TCR-α chain, and MouselincRNA0135 were found to be crucial in the pathogenesis of DCM and could thus serve as diagnostic biomarkers [49]. Another study looking for possible biomarkers in HCM patients found that serum levels of circRNAs DNAJC6, TMEM56 and MBOAT2 were downregulated in patients with HCM [50]. DNAJC6 and TMEM56 also significantly negatively predicted echocardiographic parameters for obstructive HCM. These studies illustrate the intricate machinations of lncRNA and circRNA, their ruinous or protective properties leading up to CMP and their potential role as biomarkers for different cardiomyopathy of different origins.

## Twin studies in genetic cardiomyopathies

In human genetic cardiomyopathies expression varies considerably, even among patients with the same pathogenic variant. It is postulated that a combination of genetic, epigenetic and environmental factors drives these differences, to a variable degree depending on the situation i.e. interfamilial versus intrafamilial. For instance, it is speculated that this variability may be caused by variation in genes other than the mutated one, such as by altering the clinical expression of the disease-causing mutation [51]. This assumption predicts that two carriers of such a mutation who have an identical genome should have quite similar clinical expression of the mutation they both carry. To differentiate the role of these different factors, studies in pairs of monozygotic (MZ) twins with cardiomyopathy may provide more clarity. This is a classic approach to defining the contribution of genetic and environmental factors to study variable progression of human disorders, as MZ twins have identical genome sequences.

So far, a few case reports and -series have evaluated MZ twins with cardiomyopathy [51–57]. The primary focus has been on HCM, as it is the most prevalent form and allows for the best comparison of clinical phenotypes, such as wall thickness. A case report in 2019 showed similar left ventricular wall thickness (LVWT) but different amounts of fibrosis, measured by late gadolinium enhanced magnetic resonance imaging, in a MZ twin pair with a pathogenic sarcomere mutation (c.2302G > A, p.(Gly768Arg) in the MYH7-gene) [54]. In another study, no significant heritability for interventricular septum thickness (IVSd) in HCM was found examining 11 MZ twin pairs with HCM, including 5 pairs with sarcomeric variants [51]. In contrast, a recent study demonstrated concordant morphologic findings and clinical course of identical twins with HCM, suggesting little environmental influence on clinical expression of HCM [55].

Most recently, disease progression has been studied in 11 pairs of monozygotic HCM twins [57]. Nine twin pairs were carriers of pathogenic sarcomere gene variants and two with unknown disease etiologies. The twin pairs were followed for 5 to 14 years, during which left ventricular wall thickness, left atrial diameter, and left ventricular ejection fraction were collected from echocardiograms at various time points. All 11 twin pairs were shown to have discordant morphologic features of the heart, and no notable somatic genetic variants that might explain their clinical differences were detected.

In summary, the limited studies and number of MZ twin pairs described so far point to a significant influence of non-hereditable factors, i.e. epigenetics and environment, on clinical expression of HCM. The case report by Maron et al. is an exemption, which may at least in part be explained by the non-sarcomeric CRYAB-variant found in both brothers and the high degree of shared environmental factors in this specific case.

## Environmental factors and cardiomyopathies

The main environmental factors associated with cardiovascular diseases are smoking, air pollution, alcohol, poor diet, infections, and toxins. This may have a negative impact on the clinical outcomes often seen in CMP such as HF, arrhythmias, and thromboembolisms. Interestingly, two studies have been performed with more than 400.000 participants through the UK biobank on air pollution and road noise and their effect on cardiovascular disease.

The relation between heart failure and air pollution, was studied by monitoring subjects during a mean follow-up of 10.1 years. After monitoring it was found that increased air pollution was associated with an elevated risk of heart failure. Additionally, air pollution, more specifically nitrogen dioxide, is associated with an increase in LV mass and a lower left ventricular ejection fraction in patients with DCM. The underlying mechanism appears to be air pollution's ability to rapidly affect DNA methylation, leading to hypomethylation, which is in turn associated with elevated cardiovascular disease biomarkers. Furthermore, a study found that higher levels of air pollution were negatively associated with miRNAs predicted to target anti-inflammation factors, coagulation factors and vasoconstriction factors [58].

Secondly, alcohol is known to have a direct negative impact on cardiac health. Alcoholic cardiomyopathy already was a well-known term, but the burden and interaction of alcohol with cardiomyopathy phenotype was unclear. A systematic review found a clear indication that heavy drinking over a long duration was linked to a high risk of developing CMP. However, not all individuals with a chronic high alcohol intake develop an alcoholic cardiomyopathy. In a study comparing the miRNA profile in alcohol-induced cardiomyopathy and a healthy control group, it was observed that miRNAs associated with cardiac homeostasis were altered, with miR-138 as a potentially early diagnostic marker for developing CMP [59].

Pregnancy leads to significant cardiovascular changes such as an increased cardiac output as a result of a blood volume increase, an increased heart rate, and an increase in LV mass. Mostly, these cardiovascular changes are well tolerated. However, it can lead to a CMP phenotype, in particular in women with pre-existing (asymptomatic) cardiac dysfunction and/or genetic predisposition.

Toxins is an umbrella term for a variety of risk factors such as drugs, illicit substances, and natural toxins. The anticancer drug anthracycline is known to induce cardiotoxicity. Cocaine and methamphetamines are amongst the illicit substances negatively influencing cardiovascular health. Natural toxins could be metals such as lithium, mercury, and cobalt associated with contractile abnormalities.

Poor diet or nutrition is widely known as a risk factor for cardiovascular disease [56]. Metabolic cardiomyopathy can be caused by over-nutrition. Over-nutrition could lead to cardiac insulin resistance, starting a cascade resulting in oxidative stress and ER stress changing the heart structure. A study identified five loci where DNA methylation was associated with the development of type 2 diabetes, with an increased risk of up to 3.5 times that of the general population [60].

Acute myocarditis may herald the onset of DCM or ACM. A study showed only up to 8% of patients with acute myocarditis had a genetic variant associated with DCM or ACM, thus supporting the concept environmental factors, such as infections, could trigger a CMP phenotype in genetically predisposed persons. Other hypothesis could be that a viral infection interacts with the individual's genetic material, causing an impact on the expression of genes related to cardiomyopathy.

Extensive epidemiologic studies have suggested that adult disease risk is associated with adverse environmental conditions during embryonic development, i.e. intergenerational effects, and moreover, sometimes even in second and third generation offspring, i.e. transgenerational effects. One of the best-known examples is the Dutch Hunger Winter in the western part of The Netherlands [61]. This period of famine toward the end of World War II was the consequence of a occupier-imposed food restriction. These unique features showed that prenatal exposure to famine is associated with persistent epigenetic differences in humans [62]. The association was specific for periconceptional exposure, reinforcing that very early embryonic development is a crucial period for establishing and maintaining epigenetic marks.

## New therapies targeting epigenetics

**Fig. 3:**
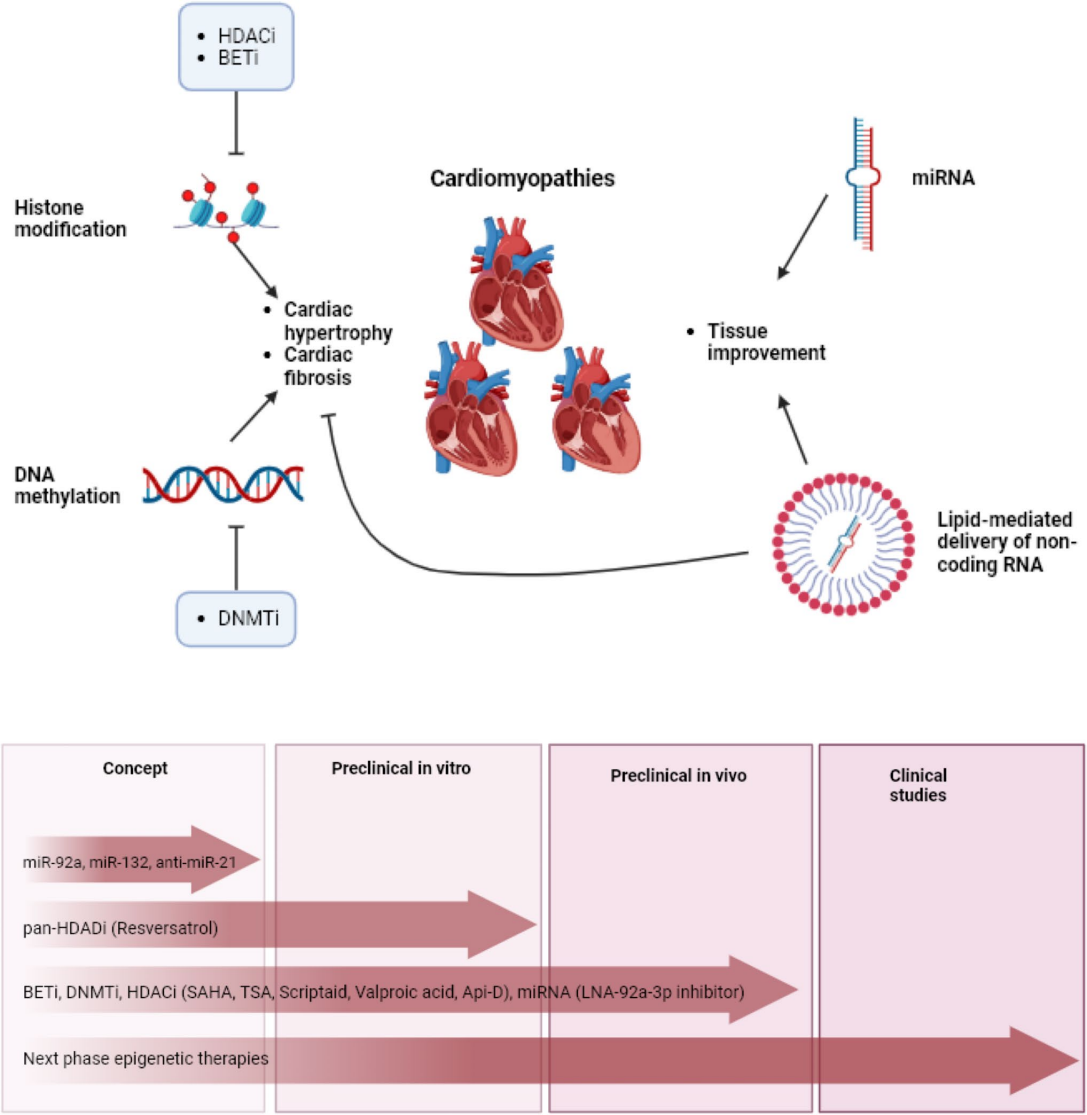
The (potential) effects of epigenetic therapies on cardiomyopathies

The plethora of knowledge gained from all the investigations not only results in knowledge on disease onset and progression, but also allows for identification of possible therapeutic targets. Current potential epigenetic targets for therapy, along with therapeutic drugs being tested in experimental and in clinical trials, are primarily focused on atherosclerosis, myocardial infarction and heart failure. However, similar pathways or specific manifestations, such as fibrosis and hypertrophy, could also be of interest for the treatment of CMPs [63]. Therapy overview is shown in the Table 1and Fig. 3.

**Table 1:** Potential epigenetic drug therapies for cardiomyopathies

Type of epigenetics	Drug	Type	Model	Therapeutic effect
**Histone modification**	Vorinostat/suberoylanilide Hydroxamic acid (SAHA) [64]	HDAC class I and II	In vivo: mice	Block cardiac fibrosis
	Trichostatin a (TSA)	HDACi class I and II	In vivo: mice	Block cardiac fibrosis
	Resversatrol [65]	Natural compound pan-HDAC inhibitor through SIRT1 activation	ln vitro: rats	Anti-inflammatory, anti-hypertrophic
	Scriptaid [66]	HDACi	In vivo: mice	Reduction cardiac fibrosis
	Valproic acid [66]	HDACi	In vivo: mice	Reduction cardiac fibrosis
	Apcidin-derivative (Api-D) [66, 67]	HDACi	In vivo: murine	Reduction cardiac fibrosis, Anti-hypertrophy
	BET inhibitor thienodiazepine (JQ1) [76]	Bromodomain and extra terminal (BET) containing protein family	In vivo: DCM patients	Reduction severe HF due to pressure overload and myocardial infarction
**DNA methylation**	5-azacytidine [68–70]	DNA methylation block	In vitro: mice; in vivo: mice	Anti-hypertrophy and reduction cardiac fibrosis
	5-aza-C [69]	DNMT inhibitor	In vivo: murine	Reduction remodeling and fibrosis
**Non-coding RNA**	LNA-92a-3p inhibitor [71]	miRNA	In vivo: HF patients and mice	Tissue improvement
	Anti-miR-21 [71]	miRNA	In vivo: mice	Reduction fibrosis
	miR-92a [71] Anti-miR-17-5p [72]	miRNA miRNA	In vivo: mice In vivo: mice	Improves angiogenesis Improves heart function
	miR-132 [71]	miRNA	In vivo: mice	Anti-hypertrophy
	Anti-miR-34a [73]	miRNA	In vivo: mice	Decrease myocardial infarction

BET = Bromodomain and extra terminal; HDAC = histone modification, histone deacetylases; miRNA = microRibonucleic acid

With regards to histone modification, HDAC inhibitors are being studied for their role in cardiac hypertrophy. One of the earliest indications of the role of HDACs in HF emerged in 2002, when Class II HDACs were shown to be stress-responsive. They were identified as regulators of the myocyte enhancer factor-2 (MEF2) transcriptional program in the heart [74]. The ability of HDAC compounds such as vorinostat/suberoylanilide hydroxamic acid (SAHA), to block cardiac fibrosis in various pre-clinical models of systolic HF is well-established [64]. Since then, SAHA along with Trichostatin A (TSA), another HDACi from the same hydroxamic class of HDACi, have been used in pre-clinical models for heart failure. In addition, resversatrol is a natural compound which has been found to have cardio protective properties, including anti-inflammatory and anti-hypertrophic, and can be seen as a pan-HDAC inhibitor [65]. An in vitro study has shown that resveratrol can attenuate cardiomyocyte hypertrophy by targeting sirtuin 1 (SIRT1), a protein of the class III NAD-dependent histone deacetylase family, and diminishing IL-6 activation [65]. Other HDACis used in pre-clinical studies include Scriptaid, Valproic acid, and Apicidin-derivative(Api-D), all exhibiting different levels of cardioprotection in mouse models of HF, including attenuation of cardiac hypertrophy and reductions in cardiac fibrosis and cardiac inflammation [66]. Via another pathway, inhibition of class I HDACs with apidicin in a murine model resulted in mTOR inhibition and subsequently attenuated cardiac hypertrophy [67].

For DNA methylation, 5-azacytidine is a demethylation medication which is being investigated. Through its blockade of DNA methylation, studies have shown that it may improve cardiac hypertrophy and reduce cardiac fibrosis by reducing the effect of tumor necrosis factor-α (TNF-α) on sarcoplasmic/endoplasmic reticulum Ca2^+^ ATPase 2a (SERCA2a), inhibiting DNA methyltransferases and blocking expression of HCM genes [68–70]. Mouse and rat models have demonstrated the utility of different DNA methyltransferase inhibitors (DNMTis) to attenuate cardiac hypertrophy and fibrosis [75]. For instance, the DNMTi 5-aza-C attenuates cardiac remodeling and cardiac fibrosis in spontaneously hypertensive rats as well as rats receiving a chronic infusion of hypertension-inducing norepinephrine [69]. Other important molecules of epigenetic therapy include the bromodomain and extra terminal (BET)-containing protein family. During severe heart failure induced by prolonged pressure overload, as well as massive anterior myocardial infarction in a mouse model, treatment with the BET inhibitor thienodiazepine (JQ1) was shown to have a therapeutic effect; therefore, early intervention with JQ1 at the very onset of pressure overload in mice prevented the development of cardiac hypertrophy and left ventricular (LV) systolic dysfunction [76].

RNA-based therapies are being developed in the form of microRNAs and lipid-mediated nanotechnologies to administer non-coding RNA, and are also being considered and tested in clinical trials [77]. In contrast to attenuating cardiac damage, these therapies aim to stimulate cardiac regeneration to prevent cardiomyocyte loss. One, miRNA-based, therapy example is LNA-92a-3p inhibitor which has shown tissue improvement after administration. MiR-92a target gene expressions of *ITGA5* and *CD93* increased upon LNA-92a-3p inhibitor treatment and demonstrated the efficiency of LNA-92a application in humans paving the way to further clinical translation [71]. ncRNAs that address key hallmarks of the cardiac remodeling process, such as anti-miR-21 in fibrosis, miR-92a in angiogenesis and miR-132 in pathological hypertrophy, may be used in the future for a more mechanistically orientated drug treatment of HF [71]. Actions of miRNAs on gene regulation networks have prompted interest in therapeutically manipulating cardiac miRNAs. Most examples in preclinical animal models utilize chemically modified antisense oligonucleotides (antagomirs) delivered by viral vectors or nonviral vectors to modulate endogenous miRNAs. For example, adenovirus-mediated upregulations of miR-21 and miR-24 have been demonstrated to have a favorable potential in the treatment of myocardial infarction [78, 79]. By contrast, downregulation of miR-17-5p with adenovirus-delivered miR-17-5p antagomir inhibits apoptosis of rat hearts, protects cardiac structure and improves heart function after acute myocardial infarction via the extracellular signal-regulated kinase (ERK) pathway [72]. The inhibition of miR-34a and/or miR-34 with locked nucleic acid modified antagomirs can decrease myocardial infarction, promote cardiac repair, reduce cardiac remodeling and protect heart function through de-repression of SIRT1, PNUTS, Notch1, protein O-fucosyltranferase 1 (Pofut1), Bcl2 and Cyclin D1 [73, 80, 81]. Epigenetics therefore represent great potential as a therapeutic approach in CMP given the widespread epigenetic modifications encountered. Although a number of pre-clinical studies have demonstrated therapeutic potential of some epigenetic mechanisms, clinical studies are still needed to evaluate their pharmacodynamic and pharmacokinetic potential. Majority of epigenetic mechanisms have yet to be assessed; therefore, additional avenues for developing epigenetic therapies are likely to be explored.

## Current challenges and future perspectives

In theory, epigenetic therapies offer the potential to control gene expression at the pre-transcriptional level and address gene dysregulation at its root. However, in practice, these therapies would face significant challenges in terms of clinical application. The primary concerns are the specificity and off-target effects. Since many proteins and enzymes involved in epigenetics can affect multiple histone or DNA sites systemically and are not specific to one cell type or organ, epigenetic drugs are prone to cause a broad spectrum of side effects. The oncological field is a testament to how difficult it can be to navigate these drugs with limitations based on their safety profiles. For example, romidepsin is a potent class I HDAC inhibitor used for cutaneous T-cell lymphoma, and though it showed highly potent anti-tumor activity, it induced detrimental alterations in electrocardiographic patterns of patients in a phase II clinical trial [82]. Some epigenetic modifications, however, such as mutations in RBM20 appear to be organ and even gene specific, providing safer options as therapeutic targets and would be interesting to further investigate.

Another limitation to consider for clinical application is the reversible nature of epigenetic modifications. As modifications are most often reversible or stem from germline mutations, the effects of epigenetic drugs may be temporary. And if temporary treatment is enough to treat a disease, aberrant epigenetic patterns may re-emerge and result in disease recurrence. Furthermore, as epigenetics function on a small scale with environmental factors having a lot of effects, the individual epigenetic landscape per person could differ greatly and might impede therapeutic effects of a specific epigenetic drug. Besides these factors, drug delivery (tissue penetrance) and pharmacokinetics against such specific drugs might also prove difficult.

The development of indirect epigenetic testing could significantly advance cardiac diagnostics and help implement epigenetics in clinical practice. Traditionally, studying epigenetic mechanisms in patients requires cardiac biopsies of the diseased heart, which are invasive and challenging procedures for the patient and practitioner. A novel method has emerged that uses hiPSC-cardiomyocytes exposed to patient blood plasma, allowing circulating proteins, miRNAs, and other molecules to induce epigenetic changes that mimic those in the patient's heart [83]. This approach provides valuable epigenetic insights without the need for cardiac biopsies. While the current study has focused only on DNA methylation in heart failure patients specifically, investigating in cardiomyopathies and with the other epigenetic mechanisms could facilitate broader clinical applications, making epigenetic testing a more practical tool in routine diagnostics.

Another important consideration for future studies is the role of sex differences in epigenetic mechanisms and therapies. Research has already shown that sex is a predictor of the prevalence, clinical presentation, and prognosis of cardiomyopathies (CMPs). The underlying causes are thought to be multifactorial. First, genes that escape X chromosome inactivation and sex-biased genetic variations contribute to differences in CMP penetrance. Second, sex hormones, such as estrogens, influence the penetrance and severity of CMPs, often favouring females [84–86]. In addition, sex-dependent epigenetic mechanisms in cardiovascular diseases are increasingly being studied. For example, one study found that methylation of the PLA2G7 promoter, a predictive biomarker for atherosclerosis, is associated with coronary heart disease in women only [87]. In contrast, reduced methylation of F2RL3, which is involved in platelet activation, was more strongly associated with cardiac mortality in males than in females [88]. However, studies specifically addressing sex differences remain limited. A systematic review from 2018 on sex differences in cardiovascular epigenetics found only 13 papers on this topic, all focused on DNA methylation [89]. Notably, the review excluded studies involving only one sex, and of those, only 14% focused on females. This highlights the need for further exploration of sex differences not only in CMP but also in cardiovascular epigenetics to uncover potential etiologies and therapeutic markers for patients.

## Conclusions

Cardiomyopathy is complex, multifactorial diseases needed to be studied from different angles. Here we have explored the correlations between genetic and epigenetic mechanisms, the role of environmental factors, available therapies based on epigenetic mechanisms, and the available evidence from twin studies and experimental models attempting to address the complexity of CMP and its potential developmental mechanisms.

DNA methylation and chromatin remodelling have been extensively studied in certain cardiovascular fields. However, their role in cardiomyopathies remains underexplored. Current research indicates their involvement in regulating gene expression, which impacts cardiac structure and function. This highlights the potential for diagnostic markers and therapeutic targets. Therefore, further investigation into these two epigenetic mechanisms of cardiomyopathies can be a new direction worth investigating in the future. Further exploration of miRNAs involvement in development of cardiomyopathies is necessary, as these molecules can be either up-or downregulated in CMPs, making them potential biomarkers. Their ability to bind specific mRNA regions and regulate gene expression offers promise for therapeutic options, but research is still limited to primarily mice studies. Animal models are challenging to study epigenetics through conservation, whereas an opportunity arises for iPSC cells to be implemented within this field. Furthermore, given the cell-specific epigenetic factors single cell analysis should be the new focus instead of bulk analyses. Additionally, challenges such as specificity and potential off-target effects, and sex differences need to be addressed. Therefore, in the future such studies are necessary before considering miRNAs implementation as novel tools for detection and treatment of cardiomyopathies in clinical practice.

An environmental trigger may serve as a second hit (“genetics loads the gun, environment pulls the trigger”), to start or worsen the development of a cardiomyopathy in a person that was already more vulnerable to cardiomyopathy due a (epi)genetic predisposition. Examples of such environmental triggers are pregnancy, chemotherapy, toxins, alcohol, and infection. This may have a negative impact on the clinical outcomes often seen in CMP such as heart failure, arrhythmias, and thromboembolisms. Monozygotic twin studies are a classic but unique clinical approach to study insights into the role of genetic, epigenetic and environmental factors. In inherited cardiomyopathies, some studies show different phenotypes and clinical outcomes in MZ twins, suggesting contribution of epigenetic and environmental factors.

Finally, epigenetics based therapies have the potential to enable significant progress in the development of next-generation therapeutics for cardiomyopathies.

## Supplementary Information

Below is the link to the electronic supplementary material.

Supplementary file1 (DOCX 75 KB)
