# Supplementary material for: Epigenetics of cardiomyopathies: the next frontier
Source: Heart Fail Rev. 2024 Nov 26;30(1):257–70. doi: 10.1007/s10741-024-10460-4 (PMC11646213; doi:10.1007/s10741-024-10460-4)
Supplement: Supplementary file 1 — Supplementary file1 (DOCX 75 KB) [file 10741_2024_10460_MOESM1_ESM.docx]

**Supplemental Table 1A.** Overview DNA Methylation.

| **Study** | **DNA Methylation** | **Study population** |
| --- | --- | --- |
| [1] Movassagh et al. | DNA methylation was significantly different in promoter CpG islands, intragenic CpG islands, gene bodies, and H3K36me3-enriched regions of the genome. DNA methylation differences were present in promoters of upregulated genes but not downregulated genes. H3K36me3 enrichment itself was also significantly different in coding regions of the genome. Specifically, abundance of RNA transcripts encoded by the DUX4 locus correlated to differential DNA methylation and H3K36me3 enrichment. | LV tissues were from patients undergoing cardiac transplantation for end stage cardiomyopathy |
| [2]Jo et al. | Alterations in epigenetic regulation (i.e., DNA methylation) have been implicated in the development of DCM. Jo et al. identified a total of 1828 differentially methylated probes (DMPs) by comparing the methylomes between 18 left ventricles and 9 right ventricles. Alterations in DNA methylation levels were observed mainly in lowly methylated regions corresponding to promoter-proximal regions, which become hypermethylated in severely affected left ventricles. | DNA samples obtained from DCM patients |
| [3] Brochet et al. | Brochet P, et al. identified 35 CCC-specific methylation markers. In addition, 33 CpGs have been identified, allowing to predict the progression of this pathology (from moderate to severe CCC), with a sensitivity of 100% and a specificity of 93%. Finally, two set of methylation markers were identified as potentially useful for Chagas disease diagnostic. The first one permit to discriminate patients with Chagas cardiomyopathy from asymptomatic patients and the second allows to predict Chagas cardiomyopathy severity stage, according to the heart ejection fraction rate. | DNA samples from Chagas disease Cardiomyopathy patients |
| [4] Kiselev et al. | The methylome analysis revealed 1755 nominally significant differentially methylated positions (DMPs), mostly hypomethylated in HCM. Based on gene ontology enrichment analysis, the majority of biological processes, overrepresented by both differentially expressed genes (DEGs) and DMP-containing genes, are involved in the regulation of locomotion and muscle structure development. The intersection of 193 DEGs and 978 DMP-containing genes pinpointed eight common genes, the expressions of which correlated with the methylation levels of the neighboring DMPs. Half of these genes (AUTS2, BRSK2, PRRT1, and SLC17A7), regulated by the mechanism of DNA methylation, were under-expressed in HCM and were involved in neurogenesis and synapse functioning. | Myocardial samples were obtained from HCM patients and as a control group aortic stenosis patients undergoing surgical interventions. |

**Supplemental Table 1B.** Overview of histone modification

| **Study** | **Histone Modification** | **Study population** |
| --- | --- | --- |
| [5]Feng et al. | Feng Y, et al. showed that DCM-enriched H3K27ac loops largely resided in conserved high-order chromatin architectures (compartments, topologically associating domains) and their anchors unexpectedly had equivalent chromatin accessibility. In addition, they show that the DCM-enriched H3K27ac loop anchors exhibited a strong enrichment for HAND1 (heart and neural crest derivatives expressed 1), a key transcription factor involved in early cardiogenesis. In line with this, its protein expression was upregulated in human DCM and mouse failing hearts. | LV tissue samples were collected from patients with DCM during surgery . LV samples from nonfailing donor hearts served as controls. |
| [6]Pagiatakis et al. | A key epigenetic enzyme that has previously been implicated in the control of cardiomyocyte homeostasis is the histone methyltransferase G9a (Ehmt2, euchromatic histone lysine methyltransferase 2), through the deposition of H3K9me2. The roles of HDACs and HATs have been widely described in cardiovascular diseases as “writers” and “erasers” of histone acetylation, in the control of the epigenetic landscape of the heart. Very little is known about the specific mechanisms of these enzymes in the pathogenesis of DCM. | - |
| [7]Ma et al. | This study outlined a potential role of the histone acetyltransferase EP300 in the regulation of the myosin binding protein C cardiac isoform (MYBPC3), which is a thick filament accessory protein of the striated muscle sarcomere A band. Mutations in MYBPC3 result in contractile defects in DCM. This study used an in vitro model knockout model of MYBPC3, MYBPC3−/−, in hiPSC-CMs (human induced pluripotent stem-cell-derived cardiomyocytes), in order to evaluate its role in contractile deficits in the myocardium. The increase in EP300 expression in MYBPC3−/− cardiac microtissues suggested a potential epigenetic mechanism via which mechanical overload in the MYBPC3 deficient tissue, leading to contractile deficits, could be a result of aberrant histone acetylation | Human induced pluripotent stem cells. |
| [8]Jimenez et al. | Their results demonstrate that chamber-specific histone modifications can result in distinct electrical phenotypes and regulation of gene expression. | - |
| [9]Spurrell et al. | Comparison with data from fetal heart tissue uncovered that hundreds of fetal genes and more than 1,600 fetal enhancers become consistently reactivated in adult heart disease. The study identified the genomic locations and activation states of this intriguing subset of disease-associated enhancers, offering an entry point for targeted experimental exploration for hundreds of genes with disease-associated activity changes. | LV tissue from up to 26 healthy controls, 18 individuals with idiopathic DCM, and five fetal hearts. |

**Supplemantory Table1C.** Overview of Chromatin Remodeling.

| **Study** | **Chromatin Remodeling** | **Study population** |
| --- | --- | --- |
| [10]Sun et al. | Sun X, et al. examined the role of Baf60c in embryonic and postnatal heart development in a Baf60c conditional knockout mouse line and showed that Baf60c is essential for cardiac growth and cardiomyocyte function at several stages of embryonic development, by regulating broad networks of genes encoding proteins essential for function of the contractile apparatus. Many of the dysregulated genes are targets of the MEF2 co-factor MYOCD, and MYOCD was identified as a BAF60c-interacting protein showing that Baf60c is an important modulator of the fundamental program of gene expression essential for cardiac structure and function. | In vivo (mice) |
| [11]Lei et al. | The study shows that BAF250a is required for the proliferation and differentiation of cardiac progenitors both in vivo and in vitro. They identified genes regulated by BAF250a/BAF complex in cardiac cells such as Nkx2.5, Mef2c, and Bmp10 and indicated that BAF250a binds to the promoter of these targets and regulates the recruitment and activity of the SWI/SNF catalytic subunit Brg1. The results suggest that the BAF250a-containing SWI/SNF complex plays a key role in regulating the chromatin accessibility of cardiac-specific genes for proper cardiac progenitor differentiation. | In vivo (mice)  In vitro mice C2C12 myoblast cells |
| [12]Vieira et al. | Here an epigenetic mechanism was reported which activates the transcriptional landscape of Wt1 promoting epicardial activity in the developing and adult heart after MI. Specifically, they identified a novel BRG1–SWI/SNF function, implicating chromatin–remodelling of the Wt1 locus as a critical event in determining the activation and fate of embryonic EPDCs. Tβ4 physically interacts with BRG1 promoting optimal Wt1 re-expression in the adult epicardium after MI. | In vivo (mice)  In vitro healthy human cell lines |
| [13]Zeng et al. | This study shows the structural mechanism of acetylated histone binding by the double PHD fingers of DPF3b. The three-dimensional solution structures and biochemical analysis of DPF3b highlight the molecular basis of the integrated tandem PHD finger, which acts as one functional unit in the sequence-specific recognition of lysine-14-acetylated histone H3 (H3K14ac). | In vitro: C2C12 mice myoblast cells |

**Supplemantory Table 1D.** Overview of non-coding RNAs.

| **Study** | **Non-coding RNAs (miRNA, circRNA, lncRNA)** | **Study population** |
| --- | --- | --- |
| [14]Gurha et al. | PKP2, encoding plakophilin 2 (PKP2), is the most common causal gene for arrhythmogenic cardiomyopathy. PKP2 deficiency leads to suppression of the E2F1 pathway and hypermethylation of the CpG sites at miR-184 promoter, resulting in downregulation of miR-184 levels. Suppression of miR-184 enhances and its activation attenuates adipogenesis in vitro. Thus, miR-184 contributes to the pathogenesis of adipogenesis in PKP2-deficient cells. | In vitro: mice Hl-1 cells and human cardiac mesenchymal progenitor cells. |
| [15]Sommariva et al. | Diagnosis of arrhythmogenic cardiomyopathy (ACM) is challenging and often late after disease onset. Using miRNAs as a potential diagnostic tool for various pathophysiology’s has gained much interest over the last decade. One pivotal study in this regard found a correlation between ACM and low plasma levels of miR-320a; this miRNA appeared to have implications in the pathogenesis of ACM. | ACM patients and healthy controls |
| [16]Cai et al. | Dysregulated lncRNA global expression patterns have been described in cardiovascular conditions and recently research has found specific lncRNAs with roles in cardiomyopathy found that lncDACH1 (Dachshund Homolog 1) regulated cardiac function through inhibition of SERCA2a in mice. Namely, lncDACH1 upregulation impaired cardiac function as lncDACH1 directly binds to SERCA2a and promotes ubiquitination-related degradation. Subsequent reduced calcium transients and cell shortening lead to cardiac dysfunction and CMP. | In vivo: human heart tissue from heart failure patients  In vivo: mice with heart failure |
| [17]Viereck et al. | Demonstrated that lncRNA-chast (cardiac hypertrophy-associated transcript) regulated cardiac hypertrophy on a cardiomyocyte level | In vivo: mice  In vitro: human stem cell-derived cardiomyocytes |
| [18]Micheletti et al. | Revealed lncRNA-wisper (Wisp2 super-inhancer-associated RNA) as a cardiac fibroblast-enriched lncRNA, regulating fibrosis after cardiac injury and correlated with fibrosis in mice and human hearts with aortic stenosis. | In vivo: murine models of myocardial infarction and human cardiac fibroblast samples. |
| [19]Yuan et al. | The newly identified conserved circRNA DICAR (diabetes-induced circulation-associated circular RNA) was found to efficiently inhibit DCM.20 Mouse models showed that DICAR insufficiency led to spontaneous cardiac dysfunction, hypertrophy and cardiac fibrosis, whereas overexpression of DICAR alleviated DCM. Additionally, expression levels of DICAR in circulating blood cells, plasma and cardiac tissue was lower in diabetic patients than healthy controls. | In vivo: diabetic mouse hearts.  In vitro: Cardiomyocytes treated with AGEs |
| [20]Sonnenschein et al. | Found that serum levels of circRNAs DNAJC6, TMEM56 and MBOAT2 were downregulated in patients with HCM.21 DNAJC6 and TMEM56 also significantly negatively predicted echocardiographic parameters for obstructive HCM. | In vivo: human HCM and healthy controls blood samples |

[1] M. Movassagh *et al.*, "Distinct epigenomic features in end-stage failing human hearts," (in eng), *Circulation,* vol. 124, no. 22, pp. 2411-22, Nov 29 2011, doi: CIRCULATIONAHA.111.040071 [pii]

10.1161/CIRCULATIONAHA.111.040071.

[2] B. S. Jo *et al.*, "Methylome analysis reveals alterations in DNA methylation in the regulatory regions of left ventricle development genes in human dilated cardiomyopathy," (in eng), *Genomics,* vol. 108, no. 2, pp. 84-92, Aug 2016, doi: S0888-7543(16)30070-2 [pii]

10.1016/j.ygeno.2016.07.001.

[3] P. Brochet *et al.*, "Blood DNA methylation marks discriminate Chagas cardiomyopathy disease clinical forms," (in eng), *Front Immunol,* vol. 13, p. 1020572, 2022, doi: 10.3389/fimmu.2022.1020572.

[4] I. Kiselev *et al.*, "Novel Genes Involved in Hypertrophic Cardiomyopathy: Data of Transcriptome and Methylome Profiling," (in eng), *Int J Mol Sci,* vol. 23, no. 23, Dec 3 2022, doi: ijms232315280 [pii]

ijms-23-15280 [pii]

10.3390/ijms232315280.

[5] Y. Feng *et al.*, "Rewiring of 3D Chromatin Topology Orchestrates Transcriptional Reprogramming and the Development of Human Dilated Cardiomyopathy," (in eng), *Circulation,* vol. 145, no. 22, pp. 1663-1683, May 31 2022, doi: 10.1161/CIRCULATIONAHA.121.055781.

[6] C. Pagiatakis and V. Di Mauro, "The Emerging Role of Epigenetics in Therapeutic Targeting of Cardiomyopathies," (in eng), *Int J Mol Sci,* vol. 22, no. 16, Aug 13 2021, doi: ijms22168721 [pii]

ijms-22-08721 [pii]

10.3390/ijms22168721.

[7] Z. Ma *et al.*, "Contractile deficits in engineered cardiac microtissues as a result of MYBPC3 deficiency and mechanical overload," (in eng), *Nat Biomed Eng,* vol. 2, no. 12, pp. 955-967, Dec 2018, doi: 10.1038/s41551-018-0280-4 [pii]

10.1038/s41551-018-0280-4.

[8] J. Jimenez and S. L. Rentschler, "Transcriptional and Epigenetic Regulation of Cardiac Electrophysiology," (in eng), *Pediatr Cardiol,* vol. 40, no. 7, pp. 1325-1330, Oct 2019, doi: 10.1007/s00246-019-02160-w [pii]

2160 [pii]

10.1007/s00246-019-02160-w.

[9] C. H. Spurrell *et al.*, "Genome-wide fetalization of enhancer architecture in heart disease," (in eng), *Cell Rep,* vol. 40, no. 12, p. 111400, Sep 20 2022, doi: S2211-1247(22)01237-2 [pii]

10.1016/j.celrep.2022.111400.

[10] X. Sun *et al.*, "Cardiac-enriched BAF chromatin-remodeling complex subunit Baf60c regulates gene expression programs essential for heart development and function," (in eng), *Biol Open,* vol. 7, no. 1, Jan 5 2018, doi: bio.029512 [pii]

BIO029512 [pii]

10.1242/bio.029512.

[11] I. Lei, X. Gao, M. H. Sham, and Z. Wang, "SWI/SNF protein component BAF250a regulates cardiac progenitor cell differentiation by modulating chromatin accessibility during second heart field development," (in eng), *J Biol Chem,* vol. 287, no. 29, pp. 24255-62, Jul 13 2012, doi: S0021-9258(20)43275-2 [pii]

M112.365080 [pii]

10.1074/jbc.M112.365080.

[12] J. M. Vieira *et al.*, "BRG1-SWI/SNF-dependent regulation of the Wt1 transcriptional landscape mediates epicardial activity during heart development and disease," (in eng), *Nat Commun,* vol. 8, p. 16034, Jul 24 2017, doi: ncomms16034 [pii]

10.1038/ncomms16034.

[13] L. Zeng, Q. Zhang, S. Li, A. N. Plotnikov, M. J. Walsh, and M. M. Zhou, "Mechanism and regulation of acetylated histone binding by the tandem PHD finger of DPF3b," (in eng), *Nature,* vol. 466, no. 7303, pp. 258-62, Jul 8 2010, doi: nature09139 [pii]

10.1038/nature09139.

[14] P. Gurha, X. Chen, R. Lombardi, J. T. Willerson, and A. J. Marian, "Knockdown of Plakophilin 2 Downregulates miR-184 Through CpG Hypermethylation and Suppression of the E2F1 Pathway and Leads to Enhanced Adipogenesis In Vitro," (in eng), *Circ Res,* vol. 119, no. 6, pp. 731-50, Sep 2 2016, doi: CIRCRESAHA.116.308422 [pii]

10.1161/CIRCRESAHA.116.308422.

[15] E. Sommariva *et al.*, "MiR-320a as a Potential Novel Circulating Biomarker of Arrhythmogenic CardioMyopathy," (in eng), *Sci Rep,* vol. 7, no. 1, p. 4802, Jul 6 2017, doi: 10.1038/s41598-017-05001-z [pii]

5001 [pii]

10.1038/s41598-017-05001-z.

[16] B. Cai *et al.*, "Long Noncoding RNA-DACH1 (Dachshund Homolog 1) Regulates Cardiac Function by Inhibiting SERCA2a (Sarcoplasmic Reticulum Calcium ATPase 2a)," (in eng), *Hypertension,* vol. 74, no. 4, pp. 833-842, Oct 2019, doi: 10.1161/HYPERTENSIONAHA.119.12998.

[17] J. Viereck *et al.*, "Long noncoding RNA Chast promotes cardiac remodeling," (in eng), *Sci Transl Med,* vol. 8, no. 326, p. 326ra22, Feb 17 2016, doi: 8/326/326ra22 [pii]

10.1126/scitranslmed.aaf1475.

[18] R. Micheletti *et al.*, "The long noncoding RNA Wisper controls cardiac fibrosis and remodeling," (in eng), *Sci Transl Med,* vol. 9, no. 395, Jun 21 2017, doi: 9/395/eaai9118 [pii]

10.1126/scitranslmed.aai9118.

[19] Q. Yuan *et al.*, "CircRNA DICAR as a novel endogenous regulator for diabetic cardiomyopathy and diabetic pyroptosis of cardiomyocytes," (in eng), *Signal Transduct Target Ther,* vol. 8, no. 1, p. 99, Mar 8 2023, doi: 10.1038/s41392-022-01306-2 [pii]

1306 [pii]

10.1038/s41392-022-01306-2.

[20] K. Sonnenschein *et al.*, "Serum circular RNAs act as blood-based biomarkers for hypertrophic obstructive cardiomyopathy," (in eng), *Sci Rep,* vol. 9, no. 1, p. 20350, Dec 30 2019, doi: 10.1038/s41598-019-56617-2 [pii]

56617 [pii]

10.1038/s41598-019-56617-2.
